# The contribution of educational inequalities to lifespan variation

**DOI:** 10.1186/1478-7954-10-3

**Published:** 2012-02-16

**Authors:** Alyson A van Raalte, Anton E Kunst, Olle Lundberg, Mall Leinsalu, Pekka Martikainen, Barbara Artnik, Patrick Deboosere, Irina Stirbu, Bogdan Wojtyniak, Johan P Mackenbach

**Affiliations:** 1 Max Planck Institute for Demographic Research, Rostock, Germany; 2 Department of Public Health, Erasmus Medical Centre, Rotterdam, The Netherlands; 3 Department of Public Health, Academic MC, University of Amsterdam, Amsterdam, The Netherlands; 4 Centre for Health Equity Studies, Stockholm University/Karolinska Institutet, Stockholm, Sweden; 5 Department of Health Sciences, Mid Sweden University, Östersund, Sweden; 6 Stockholm Centre on Health of Societies in Transition, Södertörn University, Södertörn, Sweden; 7 Department of Epidemiology and Biostatistics, the National Institute for Health Development, Tallinn, Estonia; 8 Department of Sociology, University of Helsinki, Helsinki, Finland; 9 Department of Public Health, Faculty of Medicine, University of Ljubljana, Ljubljana, Slovenia; 10 Department of Social Research, Vrije Universiteit Brussel, Brussels, Belgium; 11 Department of Monitoring and Analyses of Population Health, National Institute of Public Health-National Institute of Hygiene, Warsaw, Poland

**Keywords:** Lifespan variation, Life expectancy, Socioeconomic inequality, Education, International variation, Mortality

## Abstract

**Background:**

Studies of socioeconomic inequalities in mortality consistently point to higher death rates in lower socioeconomic groups. Yet how these between-group differences relate to the total variation in mortality risk between individuals is unknown.

**Methods:**

We used data assembled and harmonized as part of the Eurothine project, which includes census-based mortality data from 11 European countries. We matched this to national data from the Human Mortality Database and constructed life tables by gender and educational level. We measured variation in age at death using Theil's entropy index, and decomposed this measure into its between- and within-group components.

**Results:**

The least-educated groups lived between three and 15 years fewer than the highest-educated groups, the latter having a more similar age at death in all countries. Differences between educational groups contributed between 0.6% and 2.7% to total variation in age at death between individuals in Western European countries and between 1.2% and 10.9% in Central and Eastern European countries. Variation in age at death is larger and differs more between countries among the least-educated groups.

**Conclusions:**

At the individual level, many known and unknown factors are causing enormous variation in age at death, socioeconomic position being only one of them. Reducing variations in age at death among less-educated people by providing protection to the vulnerable may help to reduce inequalities in mortality between socioeconomic groups.

## Introduction

Individuals vary greatly in lifespan. For instance, comparing the age at death of European males at the individual level to that of every other male in the same country, the average difference is around 7.5 to 10.5 years, depending on the country.^a ^This variation in lifespan has many sources, including genetic factors, lifestyle factors, socioeconomic conditions, chance, etc. One of these sources, differential mortality by socioeconomic group, has been the subject of much research. A recent European cross-country comparison revealed higher death rates in lower educational groups in all 16 populations studied, with particularly large educational differences in mortality in parts of Central and Eastern Europe [1]. What is unknown, however, is the contribution of these *between-group *differences to all *between-individual *differences.

This relates to the debate sparked by the release of the World Health Report 2000 about whether lifespan (or more broadly health) inequality should be measured over individuals or groups, with the report's authors coming out in favor of the former [2-4]. By quantifying the variation of health over all individuals in a population, they contended, a more comprehensive inquiry into the extent of health inequality could be made than by conventional methods that quantify health inequalities as differences between predefined social groups. The authors further criticized methods that exclusively compared group means, speculating that different socioeconomic groups might also have different degrees of within-group variation. Indeed all studies to date have shown that groups with lower socioeconomic status have higher dispersion in their lifespan distributions in addition to their shorter mean lifespans [5-9]. Criticism of the report centered on whether individuals can replace groups as the unit of analysis. Critics feared that monitoring the full extent of between-individual variation in and of itself would not pinpoint areas requiring public health interventions [10]. Moreover, they noted that between-individual variation in health often correlates poorly with between-group socioeconomic inequalities in health [11] and reasoned that it would remove equity and human rights considerations from the study of health inequalities [12].

Although individual- and group-level approaches are indeed not interchangeable [13], it is important to recognize that differences between individuals and differences between groups are not entirely independent of one another-between-group differences make up one component of total between-individual variation in a population. Analyzing how the two are linked would serve to put between-group differences in health within a broader perspective. Lacking in the World Health Organization (WHO) report, however, was a clear method of linking between-group differences to total variation in health. In this paper we apply a method commonly used in economic research, but as of yet not attempted in the health sciences, that allows a decomposition of all between-individual variation into two components. By adopting Theil's index, total lifespan variation can be decomposed into a between- and a within-group component [14]. Using this method, we determine the contribution of differences in age at death between socioeconomic groups, in our case classified by education, to the total between-individual variation in age at death. We apply this method to 11 European countries with high-quality data.

## Data and Methods

### Creating synthetic cohort death distributions by age, sex, and education

We used census-based data assembled and harmonized as part of the Eurothine project [15]. This comprised sex-specific death counts and exposures by sex, age (aggregated into five-year age intervals), and level of education for 11 European countries (Table 1). The data included both longitudinal census-linked and cross-sectional unlinked studies. Excluded subpopulations were Åland Island from Finland, non-Swiss nationals from Switzerland, and overseas departments, students, the military, and persons born outside of France from the French data.

**Table 1:** Countries and study type included in the analysis

Country	Years^1^	Study type	Person-years of follow-up	Number of deaths	Missing education (%)
Sweden^2^	1991-2000	Longitudinal, census-linked	48 340 986	919 508	9.8

Norway	1991-2000	Longitudinal, census-linked	22 262 277	433 282	2.3

Finland^3^	1991-2000	Longitudinal, census-linked	27 550 171	473 873	0.0

Belgium	1991-1995	Longitudinal, census-linked	27 635 206	486 222	6.0

Switzerland	1991-2000	Longitudinal, census-linked	30 728 441	538 619	0.6

France^4^	1990-1999	Longitudinal, census-linked	2 720 978	43 024	0.0

Slovenia	1991-2000	Longitudinal, census-linked	10 325 537	165 423	1.3

Czech Republic	1999-2003	Cross-sectional, unlinked	30 308 765	535 264	0.0

Poland	2001-2003	Cross-sectional, unlinked	65 844 117	1 058 745	2.0

Estonia	1998-2002	Cross-sectional, unlinked	4 141 440	60 794	2.3

Lithuania	2000-2002	Cross-sectional, unlinked	6 189 927	115 803	0.5

Special remarks

^1 ^The years pertain to the years used from the Human Mortality Database matched to the Eurothine data.

^2 ^The missing education was primarily in ages above 75 (at beginning of study). We assumed the educational proportions above this age to be the same as those observed in the 70-74 age category.

^3 ^Unknown education was classified as elementary education.

^4 ^Permanent demographic sample of 1% of population.

Comparable educational levels had been created by regrouping national education schemes into four categories of the International System of Classification of Educations (ISCED): no education to completed primary education (elementary), lower secondary education, higher secondary education, and tertiary education. For three of the countries studied (Norway, Finland, and Switzerland) the two least-educated groups had to be combined by the Eurothine data collection team either because the countries' educational system did not allow for proper differentiation between the two groups or because the proportion of subjects in the lowest educational category was too low to draw meaningful conclusions. The proportion of subjects in each educational category is shown in Table 2.

**Table 2:** Proportion of subjects in each of the following educational categories by country

	Male				Female			
	**Elementary^1^**	**Lower sec**.	**Upper sec**.	**Tertiary**	**Elementary^1^**	**Lower sec**.	**Upper sec**.	**Tertiary**

Sweden	0.30	0.10	0.43	0.16	0.30	0.11	0.40	0.19

Norway		0.33	0.47	0.21		0.41	0.44	0.15

Finland		0.51	0.28	0.21		0.56	0.26	0.18

Belgium	0.44	0.18	0.21	0.16	0.53	0.16	0.18	0.13

Switzerland		0.22	0.55	0.23		0.44	0.49	0.06

France	0.47	0.06	0.35	0.12	0.57	0.09	0.24	0.09

Slovenia	0.20	0.19	0.49	0.12	0.24	0.35	0.32	0.08

Czech Rep.	0.12	0.50	0.24	0.13	0.32	0.33	0.27	0.07

Poland	0.28	0.34	0.27	0.11	0.38	0.18	0.35	0.10

Estonia	0.11	0.22	0.50	0.17	0.15	0.18	0.51	0.17

Lithuania	0.18	0.15	0.52	0.16	0.24	0.10	0.49	0.16

^1^The lowest two educational groups were combined in Norway, Finland, and Switzerland by the Eurothine team.

The census-linked studies followed individuals for 10 years between the 1990 and 2000 census rounds. Death and exposure counts occurring within this period were aggregated by the participating statistical offices into five-year age groups (ages 30 to 85+ at baseline). Since we were unable to distinguish the year of death, we assumed that all individuals who died over the study died at the midpoint, i.e., deaths to individuals aged 30 at baseline were assumed to have occurred at age 35 (32.5 for Belgium). In the census-unlinked studies, data were aggregated cross-sectionally for a few years around the 2000 census-year round (five-year age groups, ages 30 to 85+). To make the two data formats comparable, we only used ages 35+ in these studies.

To improve the precision of the age at death distribution, the national population death and exposure counts reported by single year of age in the Human Mortality Database (HMD) [16] were proportioned out to each educational group according to their corresponding shares derived from the Eurothine data for the equivalent time periods. The matching was done by country, sex, and five-year age group. We made the assumption that in the final open-aged category mortality rate ratios between educational groups were the same as those observed in the oldest preceding age category. A previous study showed this to be the case for females but risked overestimating differences for males, who were shown to have decreasing rate ratios between educational groups up to ages 90+ [17]. Sensitivity analysis revealed few differences in lifespan variation whether assuming constant or decreasing rate ratios over the oldest ages [5]. Finally, the small number of subjects surviving to the oldest ages led to some random variation in the right tail of the death distributions. To smooth the distribution, we fitted the Kannisto model of old age mortality to ages above 80, extrapolating death counts for both males and females beyond the first age with fewer than 100 male deaths [18]. More details about the data formats and the data matching procedures can be found in the recent publication by van Raalte et al. [5].

The result of this matching was sex-specific death rates by single year of age (35 to 110+) and educational level. We then used these death rates to construct male and female life tables for each educational subgroup, thus allowing comparable age distributions of deaths that were not confounded by the age structure of the educational subgroups of the real population.

### Measuring and decomposing lifespan disparity

Determining the contribution of educational inequality to total variation in lifespan requires using a measure that is decomposable into its between-group (*BG*) and within-group (*WG*) components, such that total variation = *BG + WG*. The *BG *inequality component captures the variation in subgroup average lifespans, while the *WG *component captures the average individual-level variation calculated for each of the subgroups, with both components weighted by the subgroup's population share. The contribution of the stratifying variable (in our case education) to the total variation in lifespans then is simply the *BG *component divided by the total variation.

Only a few measures of variation are additively decomposable, and of this subset we decided to apply Theil's entropy index (*T*). Theil's index was created from information theory to measure the degree of disorder in the distribution [14]. It is most widely used in studies of economic inequality but has also been applied in recent studies of lifespan variation [6,19,20]. The calculation and decomposition of this measure are presented in Additional file 1. Theil's index takes on greater values with greater dispersion in lifespans although it lacks an intuitive demographic interpretation. A value of 0 would indicate perfect equality (i.e., everyone died at the same age).

Even if measures of lifespan variation are highly correlated [21,22], they can arrive at different conclusions depending on their sensitivities to changes at different ends of the age distribution of death [6]. In particular *T *is known to be sensitive to changes in the early part of the distribution and becomes progressively less sensitive to changes at older ages [23]. We therefore decided to also calculate the variance in age at death (*V*), which is more sensitive to changes at older ages of the age at death distribution than *T*. Additionally, the variance examines absolute changes in variability (i.e., the measure is insensitive to additive changes to each individual's lifespan), while Theil's index measures relative changes in variability (i.e., the measure is insensitive to proportional changes in each individual's lifespan). The choice of measure is inherently a normative one. From a public health perspective it is clear that reducing lifespan variation by reducing premature mortality is a desirable outcome. It is less obvious whether higher lifespan variation caused by increased survivorship at old ages should be of concern. For this reason we prefer the age at death sensitivity profile of *T*. The calculation and decomposition of *V*, as well as the full results for this alternative measure are given in Additional file 1.

## Results

All countries in our study showed large educational differences in average age at death (Table 3). Differences tended to be smaller in Western Europe, where the highest-educated women typically lived 2.5 to 4 years longer than the least-educated women, and differences amounted to 5 to 7 years among men. In Central and Eastern European countries, these educational differences in life expectancy were considerably larger. Men in the Czech Republic had the largest differences: 16.5 years between the highest- and least-educated groups. These larger differences owed to the substantially poorer performance of the least-educated groups in Central and Eastern Europe. The tertiary educated lived to a more similar age in all countries. Differences were always larger for men than for women.

**Table 3:** Average age at death (conditional on survival to age 35) for each country, gender, and educational group over the period of study; "total" refers to all educational groups combined

	Male					Female				
	**Elem-entary^1^**	**Lower sec**.	**Upper sec**.	**Tertiary**	**Total**	**Elem-entary^1^**	**Lower sec**.	**Upper sec**.	**Tertiary**	**Total**

Sweden	75.8	76.8	78.1	80.6	77.5	80.8	82.3	83.0	84.7	82.1

Norway		74.5	77.0	79.4	76.5		80.4	82.5	83.9	81.6

Finland		73.0	75.1	78.1	74.4		80.3	82.1	83.2	81.1

Belgium	73.6	75.5	76.3	78.4	75.0	80.2	82.1	82.5	83.0	80.9

Switzerland		74.5	77.6	80.0	77.3		82.1	83.7	84.6	82.9

France	73.7	76.8	77.1	80.5	75.6	82.1	83.9	84.6	85.0	82.8

Slovenia	69.3	70.6	73.6	77.4	72.4	78.0	79.2	80.8	82.4	79.4

Czech Rep.	64.4	74.2	77.5	80.9	73.3	78.0	79.4	81.9	84.0	79.3

Poland	68.6	69.7	76.4	79.7	72.1	78.4	77.2	82.3	83.9	79.7

Estonia	62.6	63.7	68.0	75.6	67.8	71.7	74.9	78.1	81.7	77.7

Lithuania	63.4	63.0	70.9	76.9	69.4	72.4	73.6	82.4	84.0	79.3

^1^The lowest two educational groups were combined in Norway, Finland, and Switzerland by the Eurothine team.

Countries with large educational differences in life expectancy also tended to have higher overall levels of between-individual lifespan variation (Table 4). The differences again tended to follow regional patterns, with Western European countries having the lowest levels of lifespan variation, and some Central and Eastern European countries, particularly Estonia and Lithuania, the highest. Comparing Theil's index of lifespan variation by educational group, we see that in all countries, the higher the level of education, the less the between-individual lifespan variation within the group. The differences between countries in between-individual lifespan variation were also largest among the least-educated groups. In fact, the highest-educated groups in all countries had similar levels of lifespan variation.

**Table 4:** Theil's index of lifespan inequality (× 100) by country, gender and educational subgroup; "total" refers to the male/female total population Theil's index

	Male					Female				
	**Elem-entary^1^**	**Lower sec**.	**Upper sec**.	**Tertiary**	**Total**	**Elem-entary^1^**	**Lower sec**.	**Upper sec**.	**Tertiary**	**Total**

Sweden	1.49	1.35	1.19	0.98	1.28	1.24	1.13	1.01	0.87	1.08

Norway		1.50	1.23	1.01	1.29		1.22	0.99	0.86	1.08

Finland		1.72	1.56	1.14	1.58		1.17	0.96	0.88	1.07

Belgium	1.50	1.35	1.29	1.14	1.39	1.18	1.09	1.05	0.99	1.12

Switzerland		1.68	1.29	1.07	1.35		1.10	0.95	0.93	1.02

France	1.90	1.56	1.55	1.20	1.71	1.23	1.12	0.98	0.89	1.14

Slovenia	1.96	1.75	1.50	1.19	1.65	1.31	1.16	1.11	1.00	1.18

Czech Rep.	1.97	1.66	1.35	1.06	1.72	1.21	1.26	1.07	0.79	1.18

Poland	2.37	1.72	1.55	1.24	1.94	1.45	1.27	1.16	0.91	1.30

Estonia	3.16	2.76	2.24	1.52	2.49	3.01	2.05	1.43	0.99	1.75

Lithuania	3.33	3.01	2.39	1.60	2.72	3.07	2.21	1.57	1.08	2.07

^1^The lowest two educational groups were combined in Norway, Finland, and Switzerland by the Eurothine team.

Differences between educational groups accounted for between 1.7% to 10.9% of total variation in age at death among men, while for women between-group differences accounted for 0.6% to 9.0% of total variation (Table 5). Similar results were obtained using the *V *measure (see Additional file 1). Between-group differences explained more of the total variation in age at death in Central and Eastern Europe. This is particularly true for males in the Czech Republic, both because of the high between-group component and, as compared to other countries in its regional grouping, the low within-group component.

**Table 5:** Decomposition of Theil's index of lifespan inequality into its between-group and within-group components by country and gender

	Theil's index (× 100)		Within-group component		Between-group component		*BG *inequality as % of total	
	**Male**	**Female**	**Male**	**Female**	**Male**	**Female**	**Male**	**Female**

Sweden	1.28	1.08	1.26	1.07	0.02	0.01	1.7	1.3

Norway	1.29	1.08	1.27	1.06	0.03	0.01	2.0	1.2

Finland	1.58	1.07	1.55	1.06	0.04	0.01	2.2	1.0

Belgium	1.39	1.12	1.36	1.11	0.03	0.01	2.0	1.0

Switzerland	1.35	1.02	1.32	1.01	0.03	0.01	2.1	0.6

France	1.71	1.14	1.66	1.13	0.05	0.01	2.7	1.0

Slovenia	1.65	1.18	1.60	1.17	0.06	0.01	3.5	1.2

Czech Rep.	1.72	1.18	1.53	1.15	0.19	0.03	10.9	2.4

Poland	1.94	1.30	1.78	1.26	0.16	0.04	8.2	3.4

Estonia	2.49	1.75	2.31	1.67	0.18	0.08	7.4	4.4

Lithuania	2.72	2.07	2.49	1.88	0.24	0.19	8.6	9.0

**Figure 1:**
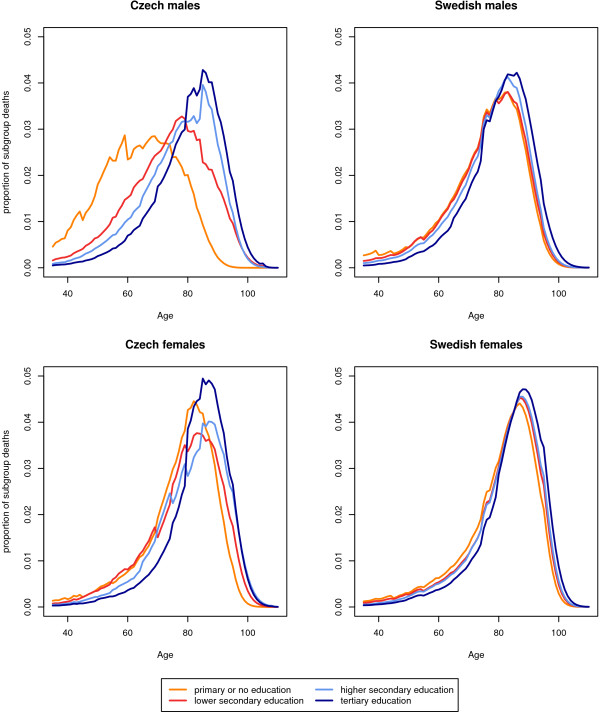
**Unsmoothed life table death distributions by educational subgroup for the Czech Republic (1999-2003) and Sweden (1991-2000)**.

Figure 1 visualizes the between-group and within-group differences in age at death for two sample countries and illustrates that most of the total variation in age at death comes from within the groups. The male Czech population has the highest contribution of the between-group component. In comparison to the Swedish population the age at death distributions are more stratified, particularly between the least-educated group and the others.

## Discussion

### Summary of results

Educational differences in age at death were substantial in all European countries but contributed only a small fraction to the total individual lifespan variation: 0.6% to 2.7% in Western Europe and 1% to 11% in Central and Eastern Europe. Less-educated groups not only had shorter mean lifespans but also had greater between-individual variation in lifespan. The gap in between-individual lifespan variation between Western Europe and Central and Eastern Europe was more evident among the least-educated groups-the tertiary-educated groups had more similar lifespan distributions in all countries.

### Evaluation of data and methods

One concern is whether, given our limited number of subgroups, we are fully capturing the educational gradient in mortality. When possible we used four subgroups, but in some countries we were restricted to three subgroups, and in others (e.g., Switzerland) the vast majority of the population fell into only two subgroups. This might have resulted in a lower than actual between-group component. To be sure that the different number of subgroups was not biasing our intercountry comparisons of the contribution of between-group inequality, we also ran the analysis for all countries with educational groups one and two combined. The reduction from four to three subgroups decreased the between-group component by 15% on average (results not shown). Using three subgroups altered the country rankings only slightly, with no rank changes for females and Poland and Lithuania trading places among males, when it came to examining the overall contribution of between-group variation to the total variation in age at death. Although more subgroups would increase the BG component, so long as we are capturing most of the linear educational gradient in mortality, we do not expect this effect to be large. Even if the between-group component were to double, it would still only explain a small fraction of individual level lifespan variation.

Education is not the only component of socioeconomic status. British studies have shown, for instance, that adding car ownership or housing tenure introduced health gradients within broad occupational categories [24-26]. Presumably, other measures of socioeconomic status would explain some of the within-educational group lifespan variation that we have found here. Although the high correlation between socioeconomic variables suggests that education would be capturing a large portion of the total socioeconomic inequality, different indicators of socioeconomic position are at times associated with different health outcomes [27-32]. Thus we expect our results to be robust for capturing the extent of the contribution of educational inequalities to lifespan variation but to underestimate the full extent of all socioeconomic inequalities.

Another concern is whether the nature of unlinked studies may introduce a numerator/denominator bias. Authorized informants may state a different educational status for the deceased than was recorded in the population census. If the deceased are reported as having a higher than attained educational level ("promoting the dead"), this would lead to overestimating mortality among the highest-educated groups [33]. However, a record linkage study for Lithuania found that unlinked estimates overestimated mortality in lower educational groups and underestimated mortality in the highest-educated groups, particularly for females [34]. We were able to compare our unlinked estimates with these linked Lithuanian data [35] (see Additional file 1). We found that the range in the average age at death between the highest- and least-educated groups was less in the linked data by 22% for males and by 34% for females. This had the effect of substantially decreasing the between-group component. As a result, the contribution of educational inequalities in age at death decreased from 7.8% to 5.0% for males and from 6.9% to 2.7% for females. While the overestimation is certainly substantial, the results from the linked data confirm a larger between-group contribution in Lithuania as compared to most Western European countries. Such a bias is also likely for Estonia, given that the two countries are post-Soviet Baltic countries that experienced similar reforms to the educational system and exhibited similar trends in unlinked age and education-specific mortality. It is more difficult to determine the direction and magnitude of bias in the Czech Republic and in Poland. The Lithuanian results are likely to be context specific and should not be generalized to other countries.

Finally, there could be problems of comparability between countries given the different study years. The unlinked studies of Central and Eastern Europe take place around the year 2000, which is on average five years later than the longitudinal census-linked studies that followed subjects for the 10-year period between the 1990 and 2000 round of censuses. Alongside changing educational compositions in the population, during this period relative inequalities in mortality between educational groups increased throughout Europe [36,37]. Some studies found that the magnitude of this widening was even greater in Central and Eastern European countries [38,39]. Thus, if we had had data for these countries for periods comparable to the longitudinal studies, we might have found smaller differences in the between-group inequality component between Eastern and Western European countries.

### Comparisons to other studies

To the best of our knowledge, we are the first to decompose individual-level variation in age at death into its between- and within-group components using Theil's index. The contribution of the between-group component that we observed is similar to American estimates made by Tuljapurkar [40], calculated by approximating the variance decomposition that we presented in Additional file 1. Morbidity researchers have decomposed the Gini coefficient or the related Health Concentration Index to determine the degree to which subgroup variation in age-standardized levels of health could be explained by socioeconomic status, a different but related question [41-43]. In these studies they found a much higher contribution from the socioeconomic component than we did. Yet it is difficult to make a direct comparison here: the distribution of age-standardized levels of health, in which many individuals self-report perfect health, differs considerably from the distribution of ages at death.

### Interpretation

Should a 1% to 11% contribution from between-group differences to the total between-individual variation in age at death be considered a large or a small amount? It is important to recognize that between-individual variation arises from many different sources, including genetic, behavioral factors, environmental conditions, and chance. These factors may in part be associated with educational level and thus vary between educational groups, but there is likely to be even more variation on many of these factors within educational groups.

We are not the first to point out that between-group differences in life expectancy account for little of the total between-individual variation. Doblhammer found that a lifespan difference of nearly half a year by month of birth explained just over 0.01% of the total variation in age at death [44]. In an additional analysis, we applied Theil's decomposition method to calculate the contribution of between-sex differences to total variation in age at death, using data from all countries of the Human Mortality Database for the year 2005. We found that the between-group component explained between 1.6% (England and Wales) and 9.9% (Russia) of total lifespan variation (results not shown). It would be interesting to run this type of analysis for risk factors such as smoking. It is also likely that lifespan variation within smoking and nonsmoking groups is larger than average differences in lifespan between the two groups. Thus we would expect a relatively small contribution from the smoking-related between-group component despite a 10-year difference in life expectancy between smokers and nonsmokers [45].

Hence it is not that between-group educational differences in mortality are small, it is more that the magnitude of all interindividual lifespan variation is tremendous. Even the large five-year advantage in life expectancy held by the highest-educated Swedish males over their least-educated counterparts acted mostly to shift the whole death distribution to higher ages (Figure 1). It did not alter the shape of the two distributions, which remained largely overlapped, owing to the much greater within-group variation.

In addition to putting inequalities in mortality between socioeconomic groups within a broader perspective, our analysis leads to some new insights into the nature of these inequalities. Educational subgroups differ not only in their mean length of life but also in the spread around that mean: the shorter life expectancy of less-educated groups concurs with a much greater variation in age at death as compared to higher-educated groups. While this inverse relationship is predicted by the compression of mortality theory [46], empirically life expectancy has been shown to be a poor predictor of lifespan variation at the macro level since the 1960s for distributions conditional upon surviving childhood [19-21,47-50]. Why mortality compression differs by educational group warrants further investigation [5,7]. Also, the larger educational inequalities in mortality in some Central and Eastern European countries can be seen to arise from the larger between-individual variation in age at death within their less-educated groups. This suggests that reduction of socioeconomic inequalities in mortality might primarily require a reduction of variability in age at death. This may require better protection of people with higher vulnerability, e.g., because of smaller personal resources or less favorable living conditions. It also requires a concerted effort to tackling causes of death that dominate at young ages, such as injuries and neoplasms [5]. The results of our analysis support the idea that a main function of modern welfare states is to provide such protection against the vicissitudes of life [51].

### Implications

Returning to the debate introduced in the introduction of this paper, it seems that individual-level variations and group-level inequalities should not be seen as competing perspectives but as interrelated phenomena. The one is embedded in the other. Our analysis illustrates the suggestion by Gakidou et al. that within-group differences are themselves interesting and substantial and a necessary complement to research into between-group inequalities [52]. But simply measuring the sum of between-group and within-group differences, which was proposed by the WHO report as an alternative measure of health inequalities, cannot replace a specific focus on measuring inequality along socioeconomic lines or any other grouping of interest such as gender, ethnicity, region, or lifestyle.

Although socioeconomic differences in mortality are but one of many factors determining when individuals die, they are often seen to be among the most important and inequitable. This is because socioeconomic inequalities are at least partly avoidable, and because they follow from inequalities in the distribution of socioeconomic resources, which themselves are often seen to be unjust [53]. Even if they contribute only a small fraction of all between-individual variations in lifespan, they are a legitimate concern for public health. What this study adds is that tackling inequalities in mortality between socioeconomic groups can also be approached through reducing variation in age at death among less-educated people by providing protection to the vulnerable.

### Notes

a) Authors' calculations of the absolute interindividual difference (the Gini coefficient of lifespans multiplied by the life expectancy) based on all European period life tables (latest year) from the Human Mortality Database [16]. More information on the calculation and interpretation of this measure are available in the paper by Shkolnikov et al. [6].

## Abbreviations

BG: Between-group inequality component; HMD: Human Mortality Database; T: Theil's index; V: Variance in age at death; WG: Within-group variation component; WHO: World Health Organization.

## Competing interests

The authors declare that they have no competing interests.

## Authors' contributions

Idea and general design of the study was conceived by AvR, AK, and JM. Data analysis was done by AvR with advice from AK. Drafts of the papers were written by AvR with advice from AK and JM. All other authors contributed data from their own countries, commented on drafts of the paper, and approved the final version of the paper.

## Supplementary Material

Additional file 1 **The file contains the following: Methods for the calculation and decomposition of Theil's index and the variance in lifespan variation, full results using the variance measure, and results comparing the usage of linked and unlinked Lithuanian data**. Click here for file
