# Supplementary material for: The contribution of educational inequalities to lifespan variation
Source: Popul Health Metr. 2012 Feb 16;10:3. doi: 10.1186/1478-7954-10-3 (PMC3299617; doi:10.1186/1478-7954-10-3)
Supplement: Additional file 1 — The file contains the following: Methods for the calculation and decomposition of Theil's index and the variance in lifespan variation, full results using the variance measure, and results comparing the usage of linked and unlinked Lithuanian data. [file 1478-7954-10-3-S1.DOC]

# Additional File

**Calculating and decomposing inequality**

Although precise calculation of both Theil’s index (*T*) and the variance (*V*) require numeric integration of the survival curve, *T* has been reasonably estimated from single year life tables according to,^2^

$T=\frac{1}{l_{a}}\sum_{x=a}^{\omega} d_{x}\left[\left(\frac{\bar{x}_{x}}{e_{a}}\right)\ln\left(\frac{\bar{x}_{x}}{e_{a}}\right)\right]$, (1)

while the *V* was estimated as,

$V=\frac{1}{l_{a}}\sum_{x=a}^{\omega} d_{x}\left(\bar{x}_{x}-e_{a}\right)^{2}$, (2)

where *a* and *ω* are respectively the youngest and oldest age intervals taken from the life table, *l_a_* is the radix of the population (taken to be the initial subgroup population size), e*_a_* is the average age at death of the population, and *d_x_* and $\bar{x}_{x}$ are respectively the life table number of deaths and the average age at death in the age interval *x* to *x+1*. The male and female all education groups combined populations were created by summing the life table deaths of all educational groups for each age interval. We calculated the lifespan measures conditional upon survival to age 35 as opposed to the measures at age 35. So in this case, rather than using the remaining life expectancy at age 35 (*e*_35_), we used the average age at death conditional upon survival to age 35 (*e*_35_ + 35).

The indices were calculated for the male and female populations, then decomposed into their between- and within-group components. Calculating between-group inequality was be done by assuming that everyone in subgroup *i* had that group’s mean age at death weighted by the subgroup’s population share (*w^i^*).

$BG^{T}=\sum_{i=1}^{n} \left[w^{i}\left(\frac{e_{a}^{i}}{e_{a}^{t}}\right)\ln\left(\frac{e_{a}^{i}}{e_{a}^{t}}\right)\right]$ (3)

$BG^{V}=\frac{1}{l_{a}}\sum_{i=1}^{n} l_{a}^{i}(e_{a}^{i}-e_{a}^{t})^{2}$ (4)

In this case *n* is the number of subgroups, $e_{a}^{i}$ refers to the average age at death conditional upon survival to age 35 for subgroup *i*, and $e_{a}^{t}$ is this average age for all education groups combined. Within-group inequality is a weighted average of the inequality levels present within each subgroup calculated by,

$WG^{T}=\sum_{i=1}^{n} \left[w^{i}T^{i}\left(\frac{e_{a}^{i}}{e_{a}^{t}}\right)\right]$ (5)

$WG^{V}=\sum_{i=1}^{n} \left[w^{i}V^{i}\right]$ (6)

where *T^i^* and *V^i^* are respectively the subgroup *i* Theil’s index of inequality and variance in lifespan.

**Appendix Table 1: Decomposition of the variance measure of lifespan inequality into its between-group and within-group components by country and gender**

|  | Variance | | Within-group component | | Between-group component | | *BG* inequality as % of total | |
| --- | --- | --- | --- | --- | --- | --- | --- | --- |
|  | Male | Female | Male | Female | Male | Female | Male | Female |
| Sweden | 144.6 | 136.9 | 142.1 | 135.0 | 2.6 | 1.9 | 1.8 | 1.4 |
| Norway | 143.3 | 134.2 | 140.2 | 132.5 | 3.1 | 1.7 | 2.2 | 1.2 |
| Finland | 166.1 | 130.8 | 162.1 | 129.5 | 4.0 | 1.4 | 2.4 | 1.0 |
| Belgium | 148.6 | 137.7 | 145.4 | 136.3 | 3.2 | 1.4 | 2.1 | 1.0 |
| Switzerland | 151.4 | 130.3 | 148.0 | 129.5 | 3.4 | 0.8 | 2.2 | 0.6 |
| France | 184.8 | 144.5 | 179.4 | 142.9 | 5.4 | 1.6 | 2.9 | 1.1 |
| Slovenia | 166.0 | 139.8 | 159.8 | 138.0 | 6.1 | 1.8 | 3.7 | 1.3 |
| Czech Rep. | 182.4 | 142.1 | 162.1 | 138.5 | 20.3 | 3.6 | 11.1 | 2.5 |
| Poland | 194.2 | 156.5 | 177.4 | 150.7 | 16.8 | 5.7 | 8.7 | 3.7 |
| Estonia | 220.4 | 193.3 | 203.3 | 184.1 | 17.1 | 9.2 | 7.7 | 4.8 |
| Lithuania | 252.8 | 239.4 | 230.2 | 216.3 | 22.6 | 23.1 | 8.9 | 9.7 |

The country rankings were exactly the same in the contribution of *BG* inequality for the two inequality measures (*T* and *V*), albeit this contribution was higher for the *V* measure.

**Appendix Table 2: Comparison of linked^*^ and unlinked^†^ datasets for Lithuania; M refers to males and F to females, BG/T is the contribution of the between-group component to individual variation**

|  | **Primary & lower sec.** | |  | **Higher sec.** | |  | **Tertiary** | |  | **Total Pop.** | |  | **Between-Group** | |  | **Within-Group** | |  | **BG/T (%)** | |
| --- | --- | --- | --- | --- | --- | --- | --- | --- | --- | --- | --- | --- | --- | --- | --- | --- | --- | --- | --- | --- |
|  | **M** | **F** |  | **M** | **F** |  | **M** | **F** |  | **M** | **F** |  | **M** | **F** |  | **M** | **F** |  | **M** | **F** |
| **Average age at death conditional upon survival to age 30** | | | | | | | | | | | | | | | | | | | | |
| Unlinked | 63.5 | 74.1 |  | 70.9 | 82.4 |  | 76.9 | 84.0 |  | 69.5 | 79.7 |  | … | … |  | … | … |  | … | … |
| Linked | 65.2 | 75.9 |  | 70.0 | 79.2 |  | 75.6 | 82.4 |  | 69.5 | 78.7 |  | … | … |  | … | … |  | … | … |
| **Theil's index x 100** | | | | | | | | | | | | | | | | | | | | |
| Unlinked | 3.19 | 2.48 |  | 2.39 | 1.57 |  | 1.60 | 1.08 |  | 2.71 | 1.92 |  | 0.22 | 0.14 |  | 2.49 | 1.78 |  | 8.2 | 7.2 |
| Linked | 2.71 | 2.05 |  | 2.28 | 1.29 |  | 1.72 | 1.08 |  | 2.43 | 1.54 |  | 0.12 | 0.04 |  | 2.31 | 1.49 |  | 5.0 | 2.7 |
| **Variance** | | | | | | | | | | | | | | | | | | | | |
| Unlinked | 253 | 252 |  | 234 | 199 |  | 179 | 141 |  | 253 | 225 |  | 21 | 17 |  | 231 | 208 |  | 8.5 | 7.7 |
| Linked | 225 | 219 |  | 215 | 151 |  | 186 | 137 |  | 225 | 176 |  | 12 | 5 |  | 213 | 171 |  | 5.2 | 2.9 |

^*^ The census-linked data cover the period 01.07.2001 – 31.12.2004, with the census having taken place on 06.04.2001. Details of the linkage procedure are described in Shkolnikov et al.^34^

^†^ The unlinked dataset (with education groups one and two combined for better comparison) cover the period 2000–2002.
